# Supplementary material for: Dynalign II: common secondary structure prediction for RNA homologs with domain insertions
Source: Nucleic Acids Res. 2014 Nov 21;42(22):13939–48. doi: 10.1093/nar/gku1172 (PMC4267632; doi:10.1093/nar/gku1172)
Supplement: SUPPLEMENTARY DATA [file supp_gku1172_nar-02021-z-2014-File012.zip › manual/RNA_class/html/class_multilign__object.html]

RNAstructure Classes: Multilign\_object Class Reference

- Main Page
- Classes
- Files

- Class List
- Class Hierarchy
- Class Members

# Multilign\_object Class Reference

Multilign\_object Class.
More...

`#include <Multilign_object.h>`

Inherited by Multifind\_object.

List of all members.

|  |  |
| --- | --- |
|  |  |
| Public Member Functions | |
|  | Multilign\_object () |
|  | Default constructor:. |
|  | Multilign\_object (const vector< vector< string > > &inputlist, const bool isrna=true, TProgressDialog \*progress=NULL) |
|  | Multilign\_object (const bool Multifind, const string &outputmultifind, const vector< string > &ctfiles, TProgressDialog \*progress=NULL, const bool isrna=true) |
|  | ~Multilign\_object () |
| int | CountBP (const int i=0, const int j=0, const double percent=0.8) const |
|  | count the number of basepairs with the lowest free energies below the percent of the miminal free energy. Dsv file is used for the counting. By default, the first dsv file in the progressive dynalign calculations is used, i.e. i = 0 and j = 0. |
| int | ProgressiveMultilign (const short int numProcessors=1, const bool Dsv=1, const bool Ali=1, const short int maxtrace=750, const short int bpwin=2, const short int awin=1, const short int percent=20, const short int imaxseparation=-99, const float gap=0.4, const bool singleinsert=true, const short int singlefold\_subopt\_percent=30, const bool local=false) |
| int | MultiTempMultilign () |
| int | WriteAlignment (const string allali="all.ali") const |
|  | calculate and output multiple alignment |
| int | GetErrorCode () const |
|  | Return an error code, where a return of zero is no error. |
| string | GetErrorMessage (const int error) const |
| int | SetMaxPairs (const int maxpairs=-1) |
| int | GetMaxPairs () const |
|  | get the value of MaxPairs |
| int | AverageLength () const |
|  | get the average length of the input sequences |
| int | SetIterations (const int it=2) |
|  | set the value of iterations |
| int | GetIterations () const |
|  | get the value of iterations |
| int | SetMaxDsv (const float maxdsvchange=1) |
|  | set the value of MaxDsv/maxdsvchange |
| float | GetMaxDsv () const |
|  | get the value of MaxDsv/maxdsvchange |
| int | GetSequenceNumber () const |
|  | get the sequence number |
| int | SetIndexSeq (size\_t indexSeq=1) |
|  | set the Index Sequence for Multilign calculation. |
| int | SetIndexSeq (const string seqname) |
|  | an overloaded function accepting a string as its parameter. |
| string | GetIndexSeq () const |
|  | return the filename of the index seq. |
| void | Randomize () |
|  | randomize the order of inputList. |
| int | AddOneInput (const string seq, const string ct, const string constraint="", const string shape="") |
|  | add one entry into inputList. |
| int | RemoveOneInput (const string seq) |
|  | remove one entry from inputList. |
| void | SetSHAPESlope (const double slope=1.8) |
|  | set the slope parameter for SHAPE |
| double | GetSHAPESlope () const |
|  | get the SHAPESlope |
| void | SetSHAPEIntercept (const double intercept=-0.6) |
|  | set the intercept parameter for SHAPE. |
| double | GetSHAPEIntercept () const |
|  | get the SHAPEIntercept. |
| void | SetTemperature (const double temp=310.15) |
|  | set the temperature to fold the sequences. |
| double | GetTemperature () const |
|  | get the temperature to fold the sequences |
| void | SetNucType (const bool isrna=true) |
|  | set the flag isRNA to be true or false. By default it is true. When it is true, RNA nearest neighbor parameters are used. |
| bool | GetNucType () const |
|  | get the type fo nucleic acid |
| int | CleanupIntermediateFiles () const |
|  | delete intermediate pairwise dsv and aout files |
| void | SetProgress (TProgressDialog \*Progress=NULL) |
|  | Provide a TProgressDialog for following calculation progress. |
| void | StopProgress () |
|  | Provide a means to stop using a TProgressDialog by assigning NULL to progress pointer. |
| TProgressDialog \* | GetProgress () const |
|  | get the progress |
| void | GetInputFilenames () |
|  | For diagnostic purpose only. Output the input sequence, ct, constraints, and SHAPE filenames to stdout. |
| void | GetPairs () |
|  | For diagnostic purpose only. Output the paired sequence filenames to stdout. |
| vector< float > | get\_energies () |
| vector< float > | get\_dGIndex () |
| vector< vector< string > > | get\_pair\_alignments () |
| Protected Attributes | |
| int | ErrorCode |
| vector< string > | input\_alignment |
| vector< string > | input\_sequences |
| vector< string > | ct\_files |
| string | output\_multifind |
| vector< float > | energies |
| vector< float > | dGIndex |
| vector< vector< string > > | pair\_alignments |
| Private Types | |
| typedef vector< string >::size\_type | vs\_index |
| typedef vector< vector< string >  >::iterator | vvs\_it |
| typedef vector< vector< string >  >::const\_iterator | vvs\_cit |
| Private Member Functions | |
| int | PairSeq1 () |
| int | PairMultifindSeq1 () |
| int | PrepInput () |
|  | This function check the legality of the input filenames and prepare the parameters for the multilign calculations. It should be called before multilign calcultions at least once and whenever something related to seq/ct changes, e.g. SetIndexSeq, AddOneInput, RemoveOneInput, Randomize, etc. |
| int | PrepMultifindInput () |
| void | ToHead (vvs\_it first, vvs\_it middle) |
|  | move the element pointed by middle before the first-pointed element. |
| int | NameDsvFiles () |
|  | name all the dsv files |
| int | NameMultifindDsvFiles () |
| int | NameAliFiles () |
|  | name all the ali files |
| int | NameMultifindAliFiles () |
| Private Attributes | |
| TProgressDialog \* | progress |
| vector< vector< string > > | inputList |
| vector< pair< vs\_index,   vs\_index > > | seqPair |
| string \*\* | dsvFiles |
| string \*\* | aliFiles |
| double | temperature |
|  | The following are the parameters for Multilign calculations. |
| int | maxPairs |
| float | maxDsv |
| int | iterations |
| bool | isRNA |
| double | SHAPESlope |
| double | SHAPEIntercept |
| Dynalign\_object \* | instance |

---

## Detailed Description

Multilign\_object Class.

The Multilign\_object class provides an entry point for the Multilign algorithm. 

---

## Member Typedef Documentation

typedef vector<string>::size\_type Multilign\_object::vs\_index `[private]`

typedef vector<vector<string> >::const\_iterator Multilign\_object::vvs\_cit `[private]`

typedef vector<vector<string> >::iterator Multilign\_object::vvs\_it `[private]`

---

## Constructor & Destructor Documentation

|  |  |  |  |  |
| --- | --- | --- | --- | --- |
| Multilign\_object::Multilign\_object | ( |  | ) |  |

Default constructor:.

|  |  |  |  |  |
| --- | --- | --- | --- | --- |
| Multilign\_object::Multilign\_object | ( | const vector< vector< string > > & | *inputlist*, |  |
|  |  | const bool | *isrna* = `true`, |  |
|  |  | TProgressDialog \* | *progress* = `NULL` |  |
|  | ) |  |  |  |

Constructor:

**Parameters:**
:   |  |  |  |
    | --- | --- | --- |
    |  | *inputlist* | is a vector of vectors of strings storing the name of the filenames. Currently, inputList is an matrix of 4 columns: col 1 is the input seq filename; col 2 is the output ct filename; col 3 is the input constraint filename; col 4 is the input SHAPE filename. Empty string is not allowed for Col 1 and Col 2; If no SHAPE or folding constraints are given, Col 3 and Col 4 for the correponding sequence are empty strings. |
    |  | *isrna* | is a bool indicating the sequences are RNA or DNA. The default of true indicates RNA. |
    |  | *progress* | is a TProgressDialog for reporting progress of the calculation to the user. The default value of NULL means that no communication is provided. |

|  |  |  |  |  |
| --- | --- | --- | --- | --- |
| Multilign\_object::Multilign\_object | ( | const bool | *Multifind*, |  |
|  |  | const string & | *outputmultifind*, |  |
|  |  | const vector< string > & | *ctfiles*, |  |
|  |  | TProgressDialog \* | *progress* = `NULL`, |  |
|  |  | const bool | *isrna* = `true` |  |
|  | ) |  |  |  |

|  |  |  |  |  |
| --- | --- | --- | --- | --- |
| Multilign\_object::~Multilign\_object | ( |  | ) |  |

---

## Member Function Documentation

|  |  |  |  |  |
| --- | --- | --- | --- | --- |
| int Multilign\_object::AddOneInput | ( | const string | *seq*, |  |
|  |  | const string | *ct*, |  |
|  |  | const string | *constraint* = `""`, |  |
|  |  | const string | *shape* = `""` |  |
|  | ) |  |  |  |

add one entry into inputList.

**Parameters:**
:   |  |  |  |
    | --- | --- | --- |
    |  | *seq* | is a string value of sequence filename to be appended1 |
    |  | *ct* | is a string value of corresponding ct filename |
    |  | *constraint* | is a string value of corresponding constraint filename. By default, it is empty, meaning no folding constraint exists |
    |  | *shape* | is string value of corresponding SHAPE filename. By default, it is empty, meaning no SHAPE exists. |

**Returns:**
:   a is int value of ErrorCode

|  |  |  |  |  |
| --- | --- | --- | --- | --- |
| int Multilign\_object::AverageLength | ( |  | ) | const |

get the average length of the input sequences

**Returns:**
:   the average length of the input sequences.

|  |  |  |  |  |
| --- | --- | --- | --- | --- |
| int Multilign\_object::CleanupIntermediateFiles | ( |  | ) | const |

delete intermediate pairwise dsv and aout files

**Returns:**
:   an int value of error code.

|  |  |  |  |  |
| --- | --- | --- | --- | --- |
| int Multilign\_object::CountBP | ( | const int | *i* = `0`, |  |
|  |  | const int | *j* = `0`, |  |
|  |  | const double | *percent* = `0.8` |  |
|  | ) |  |  | const |

count the number of basepairs with the lowest free energies below the percent of the miminal free energy. Dsv file is used for the counting. By default, the first dsv file in the progressive dynalign calculations is used, i.e. i = 0 and j = 0.

**Parameters:**
:   |  |  |  |
    | --- | --- | --- |
    |  | *i* | is an int value indicating which one in the iteration. |
    |  | *j* | is an int value indicating which iteration. |
    |  | *percent* | is threshold of double value in percentage. |

**Returns:**
:   an int of the number of basepairs counted.

|  |  |  |  |  |
| --- | --- | --- | --- | --- |
| vector<float> Multilign\_object::get\_dGIndex | ( |  | ) | `[inline]` |

|  |  |  |  |  |
| --- | --- | --- | --- | --- |
| vector<float> Multilign\_object::get\_energies | ( |  | ) | `[inline]` |

|  |  |  |  |  |
| --- | --- | --- | --- | --- |
| vector<vector<string> > Multilign\_object::get\_pair\_alignments | ( |  | ) | `[inline]` |

|  |  |  |  |  |
| --- | --- | --- | --- | --- |
| int Multilign\_object::GetErrorCode | ( |  | ) | const `[inline]` |

Return an error code, where a return of zero is no error.

This function returns and error flag that is generated during construction by RNA(const char &filename, const int type, const bool IsRNA=true) or from CalculateFreeEnergy(). An error of zero is always no error. Other codes are errors and a c-string can be fetched for the error with GetErrorMessage().

**Returns:**
:   An integer that provides the error code.

|  |  |  |  |  |  |
| --- | --- | --- | --- | --- | --- |
| string Multilign\_object::GetErrorMessage | ( | const int | *error* | ) | const |

Return error messages based on code from GetErrorCode and other error codes. 0 = no error 1000 = Error associated with sequence 1 or with a procedure, function will get message from sequence 1 (the inherited RNA class). 2000 = Error associated with sequence 2, function will get message from sequence 2 (the RNA2 class). 3000 = Errors with each sequence, function will get messages from each.

**Parameters:**
:   |  |  |  |
    | --- | --- | --- |
    |  | *error* | is the integer error code provided by GetErrorCode(). |

**Returns:**
:   A string that provides an error message or from other functions that return integer error codes.

|  |  |  |  |  |
| --- | --- | --- | --- | --- |
| string Multilign\_object::GetIndexSeq | ( |  | ) | const |

return the filename of the index seq.

**Returns:**
:   a string of index seq filename.

|  |  |  |  |  |
| --- | --- | --- | --- | --- |
| void Multilign\_object::GetInputFilenames | ( |  | ) |  |

For diagnostic purpose only. Output the input sequence, ct, constraints, and SHAPE filenames to stdout.

The following functions are used for Diagnostic purpose only //////////// Generally not needed, but for debugging input.

|  |  |  |  |  |
| --- | --- | --- | --- | --- |
| int Multilign\_object::GetIterations | ( |  | ) | const |

get the value of iterations

**Returns:**
:   the value of iterations.

|  |  |  |  |  |
| --- | --- | --- | --- | --- |
| float Multilign\_object::GetMaxDsv | ( |  | ) | const |

get the value of MaxDsv/maxdsvchange

**Returns:**
:   the value of MaxDsv/maxdsvchange.

|  |  |  |  |  |
| --- | --- | --- | --- | --- |
| int Multilign\_object::GetMaxPairs | ( |  | ) | const |

get the value of MaxPairs

**Returns:**
:   the value of MaxPairs

|  |  |  |  |  |
| --- | --- | --- | --- | --- |
| bool Multilign\_object::GetNucType | ( |  | ) | const |

get the type fo nucleic acid

**Returns:**
:   return true when it is of RNA prediction; otherwise, false.

|  |  |  |  |  |
| --- | --- | --- | --- | --- |
| void Multilign\_object::GetPairs | ( |  | ) |  |

For diagnostic purpose only. Output the paired sequence filenames to stdout.

|  |  |  |  |  |
| --- | --- | --- | --- | --- |
| TProgressDialog \* Multilign\_object::GetProgress | ( |  | ) | const |

get the progress

**Returns:**
:   the pointer to TProgressDialog

|  |  |  |  |  |
| --- | --- | --- | --- | --- |
| int Multilign\_object::GetSequenceNumber | ( |  | ) | const |

get the sequence number

**Returns:**
:   the number of input sequences

|  |  |  |  |  |
| --- | --- | --- | --- | --- |
| double Multilign\_object::GetSHAPEIntercept | ( |  | ) | const |

get the SHAPEIntercept.

**Returns:**
:   SHAPEIntercept of double value.

|  |  |  |  |  |
| --- | --- | --- | --- | --- |
| double Multilign\_object::GetSHAPESlope | ( |  | ) | const |

get the SHAPESlope

**Returns:**
:   a SHAPESlope of double value.

|  |  |  |  |  |
| --- | --- | --- | --- | --- |
| double Multilign\_object::GetTemperature | ( |  | ) | const |

get the temperature to fold the sequences

**Returns:**
:   a double value of the set temperature.

|  |  |  |  |  |
| --- | --- | --- | --- | --- |
| int Multilign\_object::MultiTempMultilign | ( |  | ) |  |

|  |  |  |  |  |
| --- | --- | --- | --- | --- |
| int Multilign\_object::NameAliFiles | ( |  | ) | `[private]` |

name all the ali files

**Returns:**
:   an int value of errorcode

|  |  |  |  |  |
| --- | --- | --- | --- | --- |
| int Multilign\_object::NameDsvFiles | ( |  | ) | `[private]` |

name all the dsv files

**Returns:**
:   an int value of errorcode

|  |  |  |  |  |
| --- | --- | --- | --- | --- |
| int Multilign\_object::NameMultifindAliFiles | ( |  | ) | `[private]` |

|  |  |  |  |  |
| --- | --- | --- | --- | --- |
| int Multilign\_object::NameMultifindDsvFiles | ( |  | ) | `[private]` |

|  |  |  |  |  |
| --- | --- | --- | --- | --- |
| int Multilign\_object::PairMultifindSeq1 | ( |  | ) | `[private]` |

|  |  |  |  |  |
| --- | --- | --- | --- | --- |
| int Multilign\_object::PairSeq1 | ( |  | ) | `[private]` |

private functions /////////////////////////////// Pair sequences for dynalign calculation

**Returns:**
:   the value of ErrorCode

|  |  |  |  |  |
| --- | --- | --- | --- | --- |
| int Multilign\_object::PrepInput | ( |  | ) | `[private]` |

This function check the legality of the input filenames and prepare the parameters for the multilign calculations. It should be called before multilign calcultions at least once and whenever something related to seq/ct changes, e.g. SetIndexSeq, AddOneInput, RemoveOneInput, Randomize, etc.

**Returns:**
:   the int value of ErrorCode.

|  |  |  |  |  |
| --- | --- | --- | --- | --- |
| int Multilign\_object::PrepMultifindInput | ( |  | ) | `[private]` |

|  |  |  |  |  |
| --- | --- | --- | --- | --- |
| int Multilign\_object::ProgressiveMultilign | ( | const short int | *numProcessors* = `1`, |  |
|  |  | const bool | *Dsv* = `1`, |  |
|  |  | const bool | *Ali* = `1`, |  |
|  |  | const short int | *maxtrace* = `750`, |  |
|  |  | const short int | *bpwin* = `2`, |  |
|  |  | const short int | *awin* = `1`, |  |
|  |  | const short int | *percent* = `20`, |  |
|  |  | const short int | *imaxseparation* = `-99`, |  |
|  |  | const float | *gap* = `0.4`, |  |
|  |  | const bool | *singleinsert* = `true`, |  |
|  |  | const short int | *singlefold\_subopt\_percent* = `30`, |  |
|  |  | const bool | *local* = `false` |  |
|  | ) |  |  |  |

The core function doing dynalign calculation and templating In case of error, the function returns a non-zero that can be parsed by GetErrorMessage() or GetErrorMessageString().

**Parameters:**
:   |  |  |  |
    | --- | --- | --- |
    |  | *Dsv* | is a boolean value indicating to output pairwise dsv files or not. It has to be set to true currently. |
    |  | *Ali* | is a boolean value indicating to output pairwise ali files or not. It has to be set to true currently. |
    |  | *maxtrace* | is the maximum number of common structures to be determined. |
    |  | *bpwin* | the the base pair window parameter, where 0 allows the structures to have similar pairs and larger windows make the structures more diverse. |
    |  | *awin* | is the alignment window parameter, where 0 allows the alignments to be similar and larger values make the alignments more diverse. |
    |  | *percent* | is the maximum percent difference in total folding free energy change above the lowest for suboptimal common structures. |
    |  | *imaxseparation* | is the maximum separation between aligned nucleotides. Values >= 0 are the traditional parameter, those below zero trigger the HMM alignment method, which is now prefered. |
    |  | *gap* | is the cost of adding gap nucleotides in the alignment in kcal/mol. |
    |  | *singleinsert* | is whether single basepair inserts are allowed in one sequence vs the other. |
    |  | *singlefold\_subopt\_percent* | is the maximum % difference of folding energy above the lowest free energy structure for pairs in single sequence folding that will be allowed in the dynalign calculation. |
    |  | *local* | is whether Dynalign is being run in local (true) or global mode (false). |
    |  | *numProcessors* | is the number of processors to use for the calculation. This requires a compilation for SMP. |

**Returns:**
:   an int that indicates an error code (0 = no error, non-zero = error occurred).

|  |  |  |  |  |
| --- | --- | --- | --- | --- |
| void Multilign\_object::Randomize | ( |  | ) |  |

randomize the order of inputList.

|  |  |  |  |  |  |
| --- | --- | --- | --- | --- | --- |
| int Multilign\_object::RemoveOneInput | ( | const string | *seq* | ) |  |

remove one entry from inputList.

**Parameters:**
:   |  |  |  |
    | --- | --- | --- |
    |  | *seq* | is a string value of sequence filename of which the entry in inputList will be removed |

**Returns:**
:   a int value of ErrorCode

|  |  |  |  |  |  |
| --- | --- | --- | --- | --- | --- |
| int Multilign\_object::SetIndexSeq | ( | const string | *seqname* | ) |  |

an overloaded function accepting a string as its parameter.

**Parameters:**
:   |  |  |  |
    | --- | --- | --- |
    |  | *seqname* | is the seq filename that will be set as the index sequence. |

**Returns:**
:   an int value of ErrorCode

|  |  |  |  |  |  |
| --- | --- | --- | --- | --- | --- |
| int Multilign\_object::SetIndexSeq | ( | size\_t | *indexSeq* = `1` | ) |  |

set the Index Sequence for Multilign calculation.

**Parameters:**
:   |  |  |  |
    | --- | --- | --- |
    |  | *indexSeq* | is a size\_t value indicating which sequence is the index sequence; by default it is the 1st one. |

**Returns:**
:   a int value of ErrorCode

|  |  |  |  |  |  |
| --- | --- | --- | --- | --- | --- |
| int Multilign\_object::SetIterations | ( | const int | *it* = `2` | ) |  |

set the value of iterations

**Parameters:**
:   |  |  |  |
    | --- | --- | --- |
    |  | *it* | is an value of int assigned to iterations. By default it is set to 2. |

**Returns:**
:   an errorcode

|  |  |  |  |  |  |
| --- | --- | --- | --- | --- | --- |
| int Multilign\_object::SetMaxDsv | ( | const float | *maxdsvchange* = `1` | ) |  |

set the value of MaxDsv/maxdsvchange

**Parameters:**
:   |  |  |  |
    | --- | --- | --- |
    |  | *maxdsvchange* | is a value of float assigned to MaxDsv. By default it is set to 1. |

**Returns:**
:   an errorcode

|  |  |  |  |  |  |
| --- | --- | --- | --- | --- | --- |
| int Multilign\_object::SetMaxPairs | ( | const int | *maxpairs* = `-1` | ) |  |

**Parameters:**
:   |  |  |  |
    | --- | --- | --- |
    |  | *maxpairs* | is int value defining how the MaxPairs will be set. By default it is set to be -1, meaning the average length of all the sequences. |

**Returns:**
:   an errorcode.

|  |  |  |  |  |  |
| --- | --- | --- | --- | --- | --- |
| void Multilign\_object::SetNucType | ( | const bool | *isrna* = `true` | ) |  |

set the flag isRNA to be true or false. By default it is true. When it is true, RNA nearest neighbor parameters are used.

**Parameters:**
:   |  |  |  |
    | --- | --- | --- |
    |  | *isrna* |  |

|  |  |  |  |  |  |
| --- | --- | --- | --- | --- | --- |
| void Multilign\_object::SetProgress | ( | TProgressDialog \* | *Progress* = `NULL` | ) |  |

Provide a TProgressDialog for following calculation progress.

**Parameters:**
:   |  |  |  |
    | --- | --- | --- |
    |  | *Progress* | is a pointer to TProgressDialog |

|  |  |  |  |  |  |
| --- | --- | --- | --- | --- | --- |
| void Multilign\_object::SetSHAPEIntercept | ( | const double | *intercept* = `-0.6` | ) |  |

set the intercept parameter for SHAPE.

**Parameters:**
:   |  |  |  |
    | --- | --- | --- |
    |  | *intercept* | is a double value assigned to SHAPEIntercept. By default, it is set to -0.6. |

|  |  |  |  |  |  |
| --- | --- | --- | --- | --- | --- |
| void Multilign\_object::SetSHAPESlope | ( | const double | *slope* = `1.8` | ) |  |

set the slope parameter for SHAPE

**Parameters:**
:   |  |  |  |
    | --- | --- | --- |
    |  | *slope* | is a double value assigned to SHAPESlope. By default, it is set to 1.8. |

|  |  |  |  |  |  |
| --- | --- | --- | --- | --- | --- |
| void Multilign\_object::SetTemperature | ( | const double | *temp* = `310.15` | ) |  |

set the temperature to fold the sequences.

**Parameters:**
:   |  |  |  |
    | --- | --- | --- |
    |  | *temp* | is a double value of temperature; by default it is set to 310.15K |

|  |  |  |  |  |
| --- | --- | --- | --- | --- |
| void Multilign\_object::StopProgress | ( |  | ) |  |

Provide a means to stop using a TProgressDialog by assigning NULL to progress pointer.

|  |  |  |  |  |
| --- | --- | --- | --- | --- |
| void Multilign\_object::ToHead | ( | vvs\_it | *first*, |  |
|  |  | vvs\_it | *middle* |  |
|  | ) |  |  | `[private]` |

move the element pointed by middle before the first-pointed element.

**Parameters:**
:   |  |  |  |
    | --- | --- | --- |
    |  | *first* | is an vector<vector<string> >iterator |
    |  | *middle* | is the same type. |

|  |  |  |  |  |  |
| --- | --- | --- | --- | --- | --- |
| int Multilign\_object::WriteAlignment | ( | const string | *allali* = `"all.ali"` | ) | const |

calculate and output multiple alignment

**Parameters:**
:   |  |  |  |
    | --- | --- | --- |
    |  | *allali* | is the output filename of multiple alignment |

**Returns:**
:   an int of error code.

---

## Member Data Documentation

string\*\* Multilign\_object::aliFiles `[private]`

vector<string> Multilign\_object::ct\_files `[protected]`

vector<float> Multilign\_object::dGIndex `[protected]`

string\*\* Multilign\_object::dsvFiles `[private]`

vector<float> Multilign\_object::energies `[protected]`

int Multilign\_object::ErrorCode `[protected]`

vector<string> Multilign\_object::input\_alignment `[protected]`

vector<string> Multilign\_object::input\_sequences `[protected]`

vector<vector<string> > Multilign\_object::inputList `[private]`

Dynalign\_object\* Multilign\_object::instance `[private]`

bool Multilign\_object::isRNA `[private]`

int Multilign\_object::iterations `[private]`

float Multilign\_object::maxDsv `[private]`

int Multilign\_object::maxPairs `[private]`

string Multilign\_object::output\_multifind `[protected]`

vector<vector<string> > Multilign\_object::pair\_alignments `[mutable, protected]`

TProgressDialog\* Multilign\_object::progress `[private]`

vector<pair<vs\_index, vs\_index> > Multilign\_object::seqPair `[private]`

double Multilign\_object::SHAPEIntercept `[private]`

double Multilign\_object::SHAPESlope `[private]`

double Multilign\_object::temperature `[private]`

The following are the parameters for Multilign calculations.

---

The documentation for this class was generated from the following files:

- F:/RNAstructure-co-from-repository/RNAstructure/RNA\_class/Multilign\_object.h- F:/RNAstructure-co-from-repository/RNAstructure/RNA\_class/Multilign\_object.cpp

---

Generated on Tue Jul 30 10:52:29 2013 for RNAstructure Classes by 
 1.5.7.1
